# Supplementary material for: Optogenetic silencing of nociceptive primary afferents reduces evoked and ongoing bladder pain
Source: Sci Rep. 2017 Nov 20;7:15865. doi: 10.1038/s41598-017-16129-3 (PMC5696510; doi:10.1038/s41598-017-16129-3)
Supplement: Supplementary file 1 — Supplementary Methods [file 41598_2017_16129_MOESM1_ESM.doc]

Optogenetic silencing of nociceptive primary afferents reduces evoked and ongoing bladder pain

Vijay K. Samineni^1,2#^, Aaron D. Mickle^1,2#^, Jangyeol Yoon^3^, Jose G. Grajales-Reyes^1^, Melanie Pullen^1^, Kaitlyn Crawford^3^, Kyung Nim Noh^3^, Graydon B. Gereau^1,2^, Sherri K. Vogt^1,2^, H. Henry Lai^2,4^, John A. Rogers^3,5,6^ and Robert W. Gereau IV^1,2^*

^1^Washington University Pain Center and Department of Anesthesiology, ^2^Washington University School of Medicine, 660 S. Euclid Ave, Box 8054, St. Louis, MO, 63110, USA; ^3^Department of Materials Science and Engineering, University of Illinois at Urbana-Champaign, Urbana, Illinois, 61801, USA. ^4^Washington University Department of Surgery - Division of Urologic Surgery, St. Louis, MO, 63110; ^5^Department of Civil and Environmental Engineering, Mechanical Engineering, Materials Science and Engineering, Northwestern University, Evanston, IL 60208, USA. ^6^Departments of Materials Science and Engineering, Biomedical Engineering, Chemistry, Mechanical Engineering, Electrical Engineering and Computer Science, and Neurological Surgery; Center for Bio-Integrated Electronics; Simpson Querrey Institute for Nano/biotechnology; Northwestern University, Evanston, IL, 60208.

^#^ Equal contributions

* Corresponding author:

Supplementary Methods:

*Immunohistochemistry:*

In order to label L6-S1 DRG neurons that innervate the bladder, we used the fluorescent retrograde labeling agent, Cholera Toxin Subunit B Alexa Fluor® 555 (Thermo Fischer Scientific, C22843). We performed 4-6 injections (total of 10µL of CTB) into the bladder wall distributed around circumference of the bladder base of SNS-Arch mice using Nanofill syringes with a 32-gauge needle (WPI). 7 days after CTB injections, animals were euthanized, and perfused with 4% paraformaldehyde (PFA) and L6-S1 DRGs were collected. Tissues were immersion fixed for 12-16 hrs in 4% PFA at 4°C, and later transferred to a 30% sucrose solution for 12-16 hours for cryoprotection. Tissues were embedded (Tissue-Tek, O.C.T. Compound, 4583) and sectioned in a cryostat. Serial 30 μm sections were collected and mounted on slides for further tissue processing. Tissues were stored at -20°C until immunohistochemistry was performed. Slides were thawed at room temperature and once dry, tissues were washed in PBS and incubated in blocking solution (10% normal goat serum/0.1% Triton-X/1x PBS) for 1 hour at room temperature. Primary antibodies (1:200 Mouse anti-CGRP, Sigma C7113; 1:200 Mouse anti-NF200, Sigma N0142; 1:1000 Rabbit anti-GFP, Thermo Fischer A11122), or IB4 (1:100, IB4 Alexa Fluor 647, Thermo Fischer I32450) were diluted in blocking solution. Slides were incubated 12-16 hours with primary antibodies of CGRP/NF200/IB4 and GFP, covered from light at 4°C. Slides were washed 3x for 10min each with PBS and incubated with secondary antibodies diluted in blocking solution for 1 hour at room temperature (1:1000 Goat anti-mouse IgG Alexa Fluor 647, Thermo Fischer A-21235; 1:1000 Goat anti-rabbit IgG Alexa Fluor 488, Thermo Fischer A11008). Slides were washed 3x for 10min each with PBS, and allowed to dry before mounting coverslips (Vectashield Hard Set, H-1400). Mounting media was allowed to dry for 2-3 hours before imaging or storage of slides at 4°C. Samples were imaged using a Leica TCS SP5 confocal microscope. The data represent a total of 3 animals and approximately 10 images per animal. Quantification of CGRP/NF200/IB4 and GFP was done blinded to the staining conditions. Once all image acquisition and analysis was finalized, the experimenter was unblinded and data were organized according to the staining for CGRP/NF200/IB4.

Whole mount staining was performed on naïve SNS-Arch mice without CTB injection. Briefly, mice were perfused with 1x PBS and bladders were removed and pinned flat to in a Sylgard dish containing cold 4% PFA. Bladders were post-fixed overnight at 4°C and then they were washed with 3x with 1x PBS and incubated for 2 hours in blocking buffer. Bladders were incubated 4-5 days in blocking buffer containing primary antibodies of (1:100 Chicken anti-GFP, Aves GFP-1020; 1:1000 Rabbit anti-βtubulin, BioLegend 802001) covered from light at 4°C. Bladders were washed 5x for 15min each with PBS and then incubated with secondary antibodies diluted in blocking solution for 2 hours at room temperature (1:1000 Goat anti-rabbit IgG Alexa Fluor 555, Thermo Fischer A21429; 1:1000 Goat anti-chicken IgG Alexa Fluor 488, Thermo Fischer A11039). Bladders were washed 5x for 15 min each with PBS, floated on to coverslips and allowed to dry before mounting coverslips (ProLong Gold antifade reagent, Molecular Probes P36934). Whole mounts were imaged using a Leica TCS SP5 confocal microscope.

*Dorsal Root Ganglion (DRG) culture*

Adult SNS-Arch mice were injected with 10µL (4-6 injections around the bladder base) DiI (ThermoFisher) into the bladder wall to allow identification of bladder-projecting sensory neurons. DRGs were dissected from these SNS-Arch mice in ice-cold Ca^2+^/Mg^2+^-free Hank’s buffered saline solution (HBSS) containing 10 mM HEPES. The tissue was digested with 45U papain (Worthington Biochemical) in HBSS+HEPES for 20 min at 37°C, washed three times with 3 ml of HBSS+HEPES at 37°C and digested in collagenase (1.5 mg/ml; Sigma) for an additional 20 min at 37°C. After the enzymatic reaction, DRGs were washed with HBSS+HEPES and mechanically dissociated by gentle trituration in Neurobasal A media (Gibco) containing 5% FBS (Life Technologies), 2 mM GlutaMAX (Life Technologies), 1×B27 supplement (Gibco), and 100 U/ml penicillin/streptomycin (Life Technologies). The DRG suspension was filtered using a 40 μm nylon filter, centrifuged (1,000g) for 3 min, resuspended and then centrifuged (1,000g) an additional 3 min. Cells were resuspended in DRG media and seeded on glass coverslips pre-coated with collagen and poly-D-lysine (Sigma). Cells were incubated at 37°C with 5% CO_2_ for 72 hours before electrophysiological recordings.

*Whole-cell electrophysiology*

Whole-cell patch clamp recordings on DRG neurons (15–30 μm in diameter) were conducted 72 hours after plating. Fire polished, filamented glass electrodes were pulled using a P-97 horizontal puller (Sutter Instrument Company) with open tip resistances ranging from 2.0–4.5 MΩ. The internal solution of the pipette contained (in mM): 120 K+ gluconate, 5 NaCl, 2 MgCl2, 0.1 CaCl2, 10 HEPES, 1.1 EGTA, 4 Na2ATP, 0.4 Na2GTP, 15 sodium phosphocreatine; pH adjusted to 7.3 using KOH, osmolarity 291 mOsm. While recording, cells were continuously perfused with external solution at room temperature containing (in mM): 145 NaCl, 3 KCl, 2.5 CaCl2, 1.2 MgCl2, 10 HEPES, 7 glucose, adjusted to pH 7.4 with NaOH. Neurons were recorded and optically stimulated with an EPC10 amplifier (HEKA Instruments) and Patchmaster software (HEKA Instruments). Optical stimulation was delivered through the microscope objective, using a custom set-up with a green (530nm) LED (M530L3; Thorlabs) coupled to the back fluorescent port of an Olympus BX-50 microscope. Light intensity of the LED at the focal plane was 10 mW/mm^2^. This was calculated using a photodiode (S120C, Thorlabs) and power meter (PM100D, Thorlabs). The series resistance of each recording was less than 20 MΩ. Neurons were voltage clamped at −60 mV and held at −60 mV for current clamp recordings. Only neurons with a resting membrane potential more negative than -35 mV were included in the data analysis.

*Visceromotor reflex behavior*

The visceromotor reflex (VMR) in female mice was quantified using abdominal electromyograph (EMG) responses. The VMR is a reliable behavioral index of visceral nociception in rodents ^1-5^. EMG responses to phasic urinary bladder distension (UBD) were recorded in lightly anesthetized mice. Mice were anesthetized with inhaled isoflurane (2% in oxygen) and silver wire electrodes were placed in the oblique abdominal muscle and subcutaneously across the abdominal wall (as a ground) to allow differential amplification of abdominal EMG signals. A lubricated, 24-gauge angiocatheter was passed into the bladder via the urethra for UBD. After surgical preparation, isoflurane was reduced to ∼1% until a flexion reflex response was present (evoked by pinching the paw), but righting reflex was absent. After the preferred level of anesthesia was attained, no adjustments were made to the isoflurane for the length of the experiment. Mice were not restrained in any fashion and body temperature was monitored and maintained at 37^o^C throughout the experiment using an overhead radiant light. UBD consisted of compressed air delivered via the transurethral catheter using a custom-made, automated distension control device (Washington University School of Medicine Electronic Shop). Phasic UBD consisted of graded distensions at pressures of 10-60 mmHg (20 s duration; 3× each pressure, 5 min inter-trial interval). EMG signals were relayed in real time using a Grass P511 preamplifier (Grass Technologies) to a PC via a WinDaq DI-720 module (Dataq Instruments), and data were exported to Igor Pro 6.05 software (Wavemetrics). Baseline EMG activity was subtracted from EMG during UBD, rectified, and integrated to obtain distension-evoked EMG responses. Distension-evoked EMG is presented as area under the curve. Fiber optic based delivery of light for VMR studies is described below. Experimenter was blinded to mouse genotype.

*Rotarod*

Locomotor activity was measured on accelerating Rotarod (Ugo Basile, Italy) to study motor coordination and balance after implantation of the optoelectronic devices over bladder as previously described ^6^. Five consecutive acceleration trials were performed with 5 min breaks separating each acceleration trial in sham and mice with bladder implants. The investigator was blinded to treatment (sham vs. implant).

*Open field test*

Open field activity was assessed using a VersaMax Animal Activity Monitoring System (AccuScan Instruments, Inc., Columbus, OH) as previously described ^7^. Before testing, mice were habituated to the test room in their home cages for 2 h. Sham and implanted mice were then placed in the open field during individual trials and allowed to freely explore after the experimenter exited the room. Open field locomotor activity was assessed by recording photobeam breaks in a chamber (42 × 42 × 30 cm, length × width × height) for 30 min. Total distance traveled was calculated for the entire chamber as well for the perimeter (outer 8-cm ring) and center (inner 26- × 26-cm square) regions.

*Supplementary References*

1 Castroman, P. & Ness, T. J. Vigor of visceromotor responses to urinary bladder distension in rats increases with repeated trials and stimulus intensity. *Neurosci Lett* **306**, 97-100 (2001).

2 Ness, T. J. & Elhefni, H. Reliable visceromotor responses are evoked by noxious bladder distention in mice. *The Journal of urology* **171**, 1704-1708, doi:10.1097/01.ju.0000116430.67100.8f (2004).

3 Ness, T. J., Lewis-Sides, A. & Castroman, P. Characterization of pressor and visceromotor reflex responses to bladder distention in rats: sources of variability and effect of analgesics. *The Journal of urology* **165**, 968-974 (2001).

4 Ness, T. J., Randich, A. & Gebhart, G. F. Further behavioral evidence that colorectal distension is a 'noxious' visceral stimulus in rats. *Neurosci Lett* **131**, 113-116 (1991).

5 Crock, L. W. *et al.* Metabotropic glutamate receptor 5 (mGluR5) regulates bladder nociception. *Molecular pain* **8**, 20, doi:10.1186/1744-8069-8-20 (2012).

6 Park, S. I. *et al.* Soft, stretchable, fully implantable miniaturized optoelectronic systems for wireless optogenetics. *Nat Biotechnol* **33**, 1280-1286, doi:10.1038/nbt.3415 (2015).

7 Montana, M. C. *et al.* The metabotropic glutamate receptor subtype 5 antagonist fenobam is analgesic and has improved in vivo selectivity compared with the prototypical antagonist 2-methyl-6-(phenylethynyl)-pyridine. *J Pharmacol Exp Ther* **330**, 834-843, doi:10.1124/jpet.109.154138 (2009).
